# Peripheral genetic structure of *Helicoverpa zea* indicates asymmetrical panmixia

**DOI:** 10.1002/ece3.2106

**Published:** 2016-04-06

**Authors:** Mathew Seymour, Omaththage P. Perera, Howard W. Fescemyer, Ryan E. Jackson, Shelby J. Fleischer, Craig A. Abel

**Affiliations:** ^1^ Southern Insect Management Research UnitUSDA‐ARSStonevilleMississippi38776; ^2^ Department of BiologyThe Pennsylvania State UniversityUniversity ParkPennsylvania16802; ^3^ Department of EntomologyThe Pennsylvania State UniversityUniversity ParkPennsylvania16802; ^4^ Corn Insects and Crop Genetics Research UnitUSDA‐ARSAmesIowa50011; ^5^ Present address: Molecular Ecology and Fisheries Genetics LaboratorySchool of Biological SciencesBangor UniversityDeiniol RoadBangorLL57 2UWUK; ^6^ Present address: Syngenta Crop Protection410 South Swing RoadGreensboroNorth Carolina27409

**Keywords:** DAPC, *Helicoverpa*, heliothine, moth, population genetics, stable isotopes

## Abstract

Seasonal climatic shifts create peripheral habitats that alternate between habitable and uninhabitable for migratory species. Such dynamic peripheral habitats are potential sites where migratory species could evolve high genetic diversity resulting from convergence of immigrants from multiple regionally distant areas. Migrant populations of *Helicoverpa zea* (Boddie) captured during two different seasons were assessed for genetic structure using microsatellite markers and for host plant type using stable carbon isotope analysis. Individuals (*N* = 568) were genotyped and divided into 13 putative populations based on collection site and time. Fixation indices (*F*‐statistics), analysis of molecular variance (AMOVA), and discriminant analysis of principal components (DAPC) were used to examine within and among population genetic variation. Mean number of alleles per locus was 10.25 (± 3.2 SD), and allelic richness ranged from 2.38 to 5.13 (± 3.2 SD). The observed and expected heterozygosity ranged from 0.07 to 0.48 and 0.08 to 0.62, respectively. Low *F*_ST_ (0.01 to 0.02) and high *F*_IS_ (0.08 to 0.33) values suggest captured migrants originated from breeding populations with different allele frequencies. We postulate that high genetic diversity within migrant populations and low genetic differentiation among migrant populations of *H. zea* are the result of asymmetrical immigration due to the high dispersal and reproductive behavior of *H. zea*, which may hinder the adaptation and establishment of *H. zea* to peripheral habitat. These findings highlight the importance of assessing peripheral population structure in relation to ecological and evolutionary dynamics of this and other highly reproductive and dispersive species.

## Introduction

Migration is the primary mechanism species use to avoid competition, find resources and, in sexual systems, locate mates (Dingle and Drake 2007). Several species have evolved long distance, seasonal, migratory characteristics, which are generally prompted by environmental cues (Alerstam et al. 2003; Dingle and Drake 2007). Seasonal migratory species are ecologically important in providing ecosystem services (e.g., Kremen et al. 2007) and causing economic damage due to spreading disease and destruction of agricultural products. Assessing the population‐level process associated with seasonal migrant recolonization and invasion is necessary for understanding impacts on biodiversity (Hughes et al. 2008), developing conservation planning, and species management strategies.

Immigration is a fundamental process influencing seasonal population establishment and structure. Successful establishment of seasonal, peripheral populations at the extent of species’ geographic range is influenced by the genetic diversity of the local peripheral population (Slatkin 1987) and the gene flow (i.e., immigration) (Kolbe et al. 2004) and propagule pressure (Lockwood et al. 2005) of the colonizing population(s). If gene flow or propagule pressure of colonizing population(s) to a peripheral population is low, establishment may be hindered due to resulting low abundance (i.e., fewer propagules) and low genetic diversity resulting in lower adaptive potential or Allee effects (Lee 2002; Bock et al. 2015). Reduced adaptive potential can develop from low propagule pressure and gene flow resulting in reduced genetic diversity, which allows other evolutionary processes, such as drift, to reduce genetic diversity and for deleterious genes to be maintained longer. Alternatively, if immigration of colonizers is high enough to promote admixture and increase standing genetic variation, locally adaptive phenotypes are more likely to be selected and proliferate allowing establishment and expansion to occur (Lenormand 2002). However, if gene flow is too high, such that admixture is dominated by colonizing migrants compared to local genotypes, adaptation for the peripheral habitat may be hindered (due to influx of less fit genotypes; Verhoeven et al. 2010). Poor adaptation of immigrants for the peripheral habitat can in turn result in localized extinction, especially for extreme environments (Bridle and Vines 2007).

Migratory immigration and peripheral population structure can also be inferred from stable isotopic analysis of animal tissue, including insect wings (Jackson et al. 2006; Head et al. 2010). Carbon *δ* ^13^C, particularly, has been used to track the origin of several herbivorous insect species (Gould et al. 2002; Jackson et al. 2006; Flockhart et al. 2013). Carbon isotope values of plants depend on the photosynthetic pathway during uptake such that *δ* ^13^C ratios differ between C3, C4, and intermediary plants (Abelson and Hoering 1961; Hatch and Slack 1970; Monson et al. 1984). Herbivores’ carbon isotopic values, in turn, reflect the plants they feed on and thus may indicate separate source populations related to immigrant versus local populations (Gould et al. 2002; Head et al. 2010).

*Helicoverpa zea* (Bodie) (bollworm, corn earworm) is a polyphagous pest, feeding on a wide range of C3 (e.g., tomatoes and cotton) and C4 (e.g., corn and sorghum) plant species (Kennedy and Storer 2000). While estimates of economic damage due to *H. zea* are currently unknown, the estimated cost of control and yield loss due to damage in cotton and soybean in the southern United States in 2013–2014 exceeded $10 per acre (Musser et al. 2014; Williams 2014). Preference for C3 or C4 plants has been documented for *H. zea* and reflects larval feeding habits and may indicate the geographic sources of immigrating populations by relating isotopic patterns to crop type abundances (Gould et al. 2002). *Helicoverpa zea* are capable of high reproductive rates with females typically laying between 500 and 1000 eggs (Fye and McAda 1972), and up to 3000 eggs within a 8‐ to 10‐day period (Quaintance and Brues 1905). After pupal emergence, adult *H. zea* will facultatively migrate long distances, with suspected overnight migrations upwards of 400 km, aided by low‐level jet streams (Westbrook and López 2010). *Helicoverpa zea* primarily overwinters as diapausing pupae, which burrow into the soil as larva. However, *H. zea* is not currently known to overwinter north of the 40th parallel (Blanchard 1942; Hardwick 1965, 1996), due to lack of adaptation for cold temperatures (Blanchard 1942; Morey et al. 2012). Several generations of *H. zea* occur during late spring through early fall, depending on the geographic region, with 4 to 8 generations occurring in southern populations and 1 to 2 generations occurring in peripheral, northern populations due to cold intolerance (Quaintance and Brues 1905; Morey et al. 2012). Expansion of the winter range of *H. zea* could lead to significant changes (e.g., earlier, greater) in seasonal time of pest presence, population levels, and degree of crop damage it causes in northern latitudes, which typically do not observe infestation until late summer (Blanchard 1942; Morey et al. 2012). Despite the high impact on agriculture, little is known regarding the genetic diversity and the potential impacts on range expansion of this species.

Here, we used microsatellite loci to assess genetic variation of 13 putative, migrant, peripheral populations of *H. zea* immigrating to seasonal habitat north of the known overwintering range (Morey et al. 2012). Putative populations are described below and are based on known *H. zea* migration behavior (Fitt 1989). We assessed the extent of genetic structure attributed to immigrating populations of *H. zea* to get insights into the underlying evolutionary, ecological, and demographic processes. Additionally, we assessed differences in colonizer sources using stable carbon isotope analysis. Specifically, we aimed to infer the extent of putative migratory genetic structure, and the potential implications for the geographic structure of *H. zea*. Alternatively, observation of random genetic variation would suggest asymmetrical gene flow or geographically admixed populations. Overall, genetic structure is characterized in relation to the dispersal dynamics of an important, but generally overlooked insect species, which has direct implications for land management and dispersal dynamics of other similarly high dispersal and reproductive species.

## Methods

### Sampling

**Figure 1:**
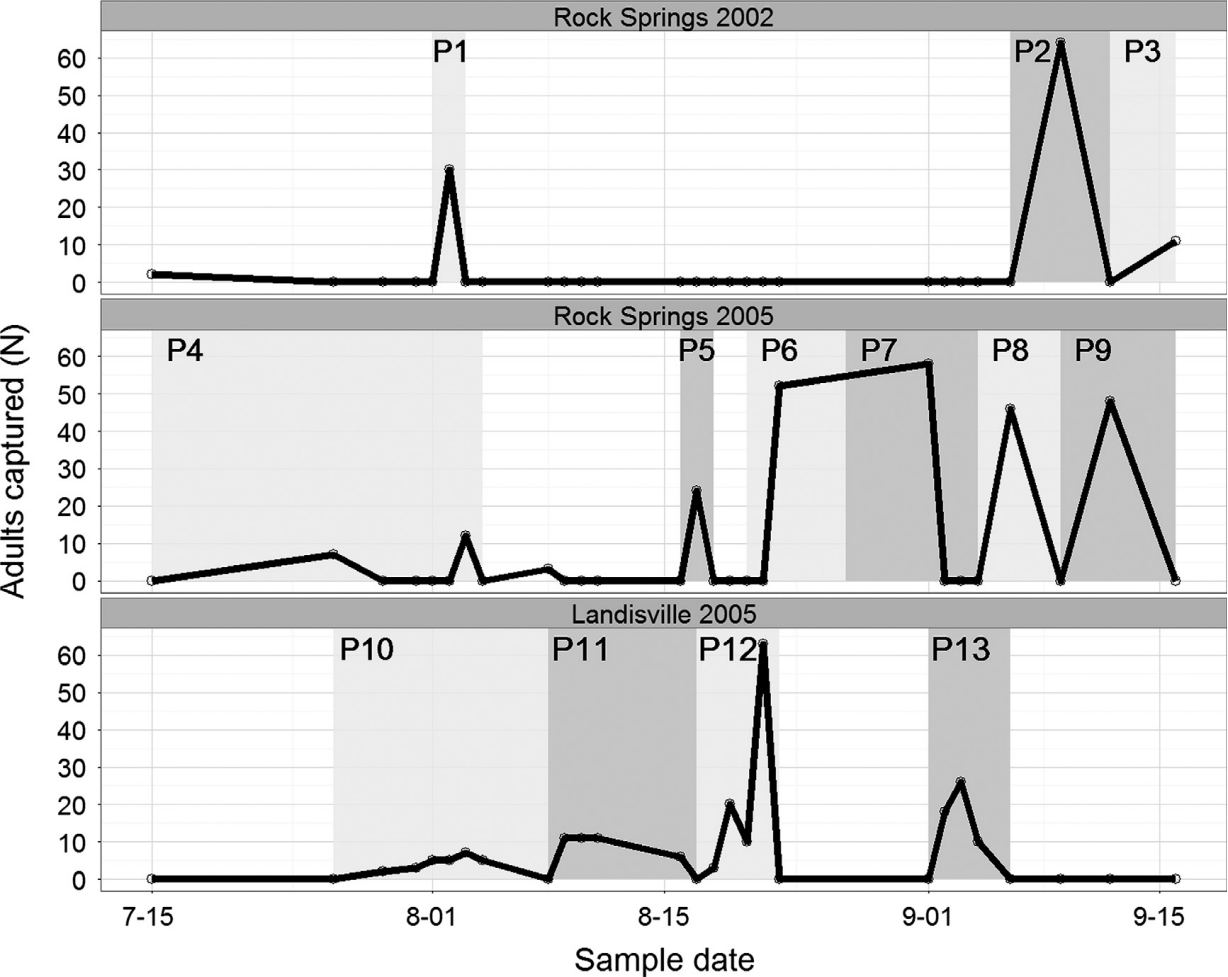
Adults captured (N) in pheromone traps from 26 July to 09 September (sample date), shown as month–day, for *Helicoverpa zea* sampled from Landisville, PA in 2005, and Rock Springs, PA in 2002 and 2005. The numbers depict putative populations (P1‐P13) of new migrants entering the respective sampling sites.

Adult *H. zea* were sampled from July through September during 2002 and 2005 near the Russel E. Larson Research and Education Center at Rock Springs, PA (40°42′38.1″N 77°57′52.2″W) and from July through September 2005 at the Southeast Research Center at Landisville, PA (40°07′04.7″N 76°25′30.5″W; Fig. 1). Adults were trapped using Hartstack pheromone traps (Hartstack et al. 1979) positioned near corn fields, which is a preferred food source for *H. zea*. All trapped individuals (*N* = 576) were collected and stored at −20°C for additional laboratory analysis. Wings dissected from each individual were preserved in 70% ethanol, and genomic DNA from thorax and head of each individual extracted using the Qiagen DNeasy Kit (Valencia, CA) at Penn State were sent to the USDA‐ARS Southern Insect Management Research Unit (SIMRU) for stable carbon isotope analysis and for microsatellite genotyping, respectively.

### Stable carbon isotope analysis

The distal half of a forewing from each moth was cut into small pieces and placed into a 5 × 9 mm tin capsule that was tightly folded. Conversion of wing tissue in each tin capsule to CO_2_ was done at SMRT using micro‐Dumas combustion using a Costech ECS4010 Elemental Analyzer (Costech Analytical Technologies Inc., Valencia, CA) coupled to a Thermo Finnigan Delta plus Advantage Mass Spectrometer using a Conflo II Interface (Thermo Scientific, Waltham, MA). The isotope standard reference material was bovine muscle powder, from the National Institute of Standards and Technology (NIST‐RM 8414). Moths were then determined to either feed primarily on C3 (*δ* ^13^C ≤ −20) or C4 (*δ* ^13^C ≥−15) plants based on previous assessment of stable carbon isotope analysis of *H. zea* (Head et al. 2010).

### Microsatellite analysis

Prior to microsatellite genotyping, DNA samples were quantified using the Quanti‐iT PicoGreen assay kit (Invitrogen, Carldbad, CA) for double‐stranded DNA and then adjusted to 20 ng/*μ*L. A set of 12 polymorphic microsatellite markers previously developed for *H. zea* was used to analyze the DNA samples (Perera et al. 2007). Polymerase chain reactions (PCR) for each sample included 1 *μ*L of DNA, 0.4 pM forward primer, 1.2 pM reverse primer, 1.2 pM 6‐carboxyfluorescein (6‐FAM)‐labeled universal primer, 1 *μ*L of 10 mM dNTP mix, 0.5 *μ*L of 10× titanium *Taq* polymerase buffer, 0.1 *μ*L of titanium *Taq* polymerase (BD Bioscience, San Jose, CA), and 1.6 *μ*L of DNase free water for a 5 *μ*L reaction. Cycling conditions included an initial denaturation step for 2 min at 95°C, an initial annealing step of 1 min at 60°C, followed by 30 denaturing and annealing cycles (15 sec at 95°C, 15 sec at 60°C, 30 sec at 72°C). Reactions were then diluted 1/9 and amplicons separated on an ABI 3700xl genetic analyzer with ROX‐labeled markers. Peaks were scored as genotypes using the software GeneMapper (Applied Biosystems, Inc., Foster City, CA) and confirmed manually.

### Genetic structure

All loci were checked for null alleles and allelic drop out using MICROCHECKER 2.2.3 (van Oosterhout et al. 2004). Individuals missing three or more loci were removed from the analysis. All additional genetic and statistical analyses were performed using the program R version 3.2.1 (R Development Core Team 2012). Markers were tested for linkage disequilibrium using the index of association (IA) and 999 permutations (Brown et al. 1980) implemented in the package poppr (Kamvar et al. 2014). Deviations from Hardy–Weinberg equilibrium (HWE) were assessed per loci across all populations using an exact test, based on Monte Carlo permutations, with 999 permutations, implemented in the package pegas (Paradis 2010). Significant levels for multiple comparisons of loci across samples were adjusted using a sequential Bonferroni correction (Rice 1989). The package hierfstat (Goudet 2005) was used to calculate expected (*H*_E_) and observed (*H*_O_) heterozygosity, within (*F*_IS_) and among (*F*_ST_) population fixation indices, pairwise *F*_ST_ and allelic richness (Ar).

Discriminant analysis of principal components (DAPC) (Jombart et al. 2010) was used as implemented in the R package adegenet (Jombart 2008) to determine population genetic structure of *H. zea* using (1) a set of prior putative *H. zea* populations, (2) assuming no prior population assignment, and (3) C3 versus C4 plant preference. Prior putative populations were determined as peak abundance periods during the collection periods, which signaled recent mass immigration of *H. zea* (Fig. 1) (Fitt 1989). Trapped moths, especially from earlier months when there are no local individuals present, are expected to be immigrant individuals. Later months may include local individuals, which may include progeny from earlier migrants as 1 to 2 generations do occur in Pennsylvania per year (Quaintance and Brues 1905; Morey et al. 2012). In total, 13 putative populations were assigned with populations sizes ranging from 11 to 95 per grouping, with an overall mean population size of 43. The nonprior population assignment DAPC analysis was used to evaluate number of clusters (K) between 2 and 20 for *H. zea*. Bayesian information criterion (BIC) was then used to evaluate the relevance of different *K* values to population structure. Assignment values for the selected number of clusters were then generated for each individual using DAPC (Jombart et al. 2010). DAPC first transforms the data using principal components analysis, which ensures that the variables are not correlated and that the number of variables is smaller than the number of individuals. Then, discriminant analysis partitions the variance into among‐ and within‐group components, maximizing separation between groups. DAPC does not assume a population genetics model and it is not constrained by Hardy–Weinberg or linkage equilibrium assumptions, making it a robust method to test for genetic differentiation. The DAPC findings were complemented by use of the program STRUCTURE to perform a Bayesian clustering analysis (Pritchard et al. 2000) to assess the possible occurrence of 1 to 13 distinct populations (*K* = 1 to *K* = 13; used 100,000 burn in and an additional 100,000 Markov chains). Analysis of molecular variance (AMOVA) was performed using Arlequin v3.5 (Excoffier and Lischer 2010) by partitioning populations into groups relative to the large peak trap catch that occurred in 2005 at Rock Springs and Landisville, PA (prepeak trap catch: P1, P4, P5, P10, P11; peak trap catch: P6, P7, P12; postpeak trap catch: P2, P3, P8, P9, P13), collection site and year, and host plant type (C3 or C4). The significance of the components of locus‐by‐locus AMOVA was tested with 10,000 permutations.

## Results

### Stable isotope analysis

**Figure 2:**
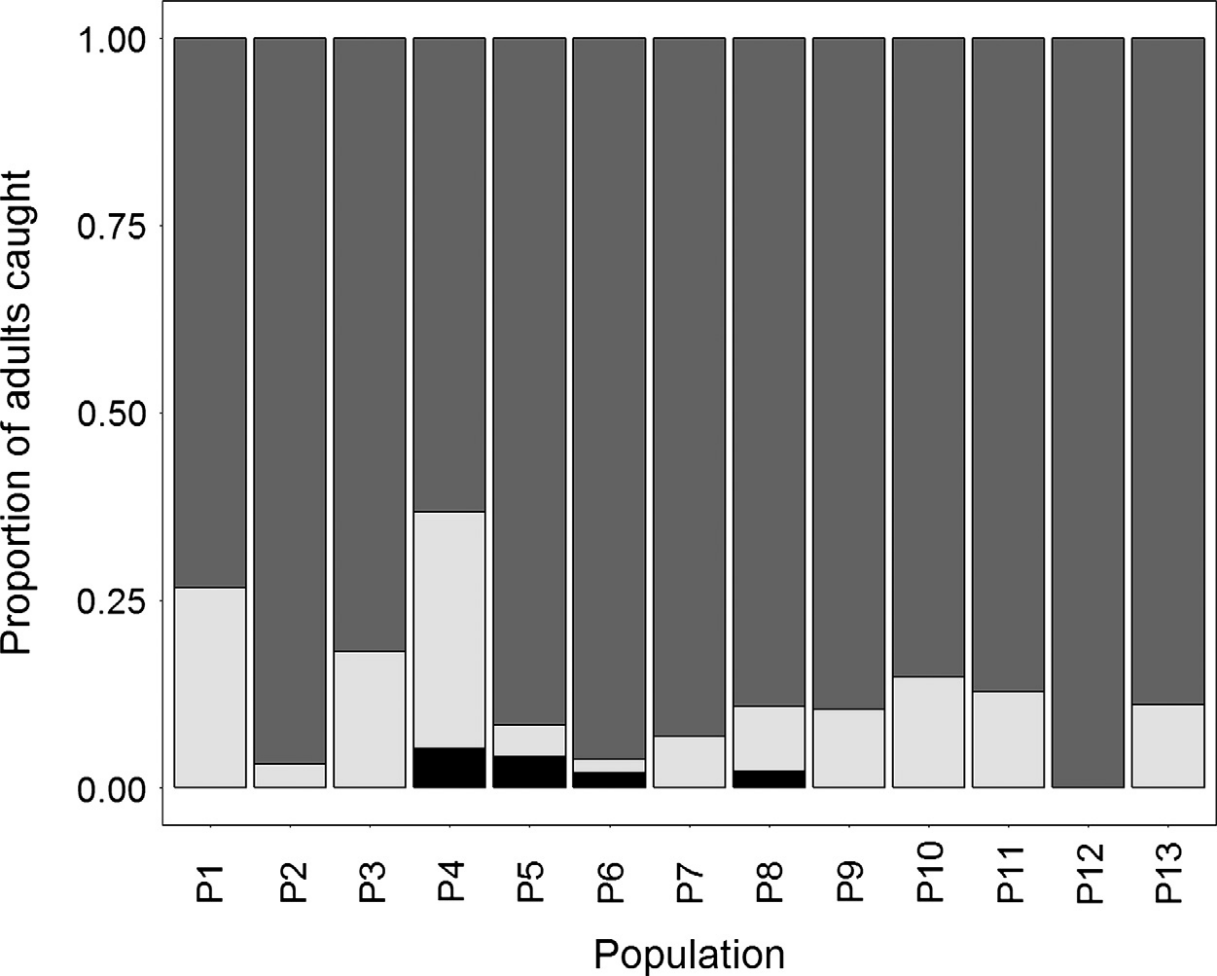
Proportion of *Helicoverpa zea* that developed on C4 (gray) C3 (light gray) or unknown (black) plants per putative population (see Fig. 1), based on stable carbon isotope analysis. Sample sizes for each population are given in Table 2.

For all 568 adults genotyped, irrespective of putative population assignment, 90.84% had *δ* ^13^C >−15 suggesting they developed on C4 host plants. Only 8.45% had *δ* ^13^C <−20, which suggested utilization of C3 host plants as larvae, regardless of sampling location. Host plant type could not be determined in only 4 individuals whose *δ* ^13^C was between −15 and −20. For adults across each putative population (Fig. 1), an average of 87% (± 10% SD) developed on C4 host plants compared to 12% (± 9% SD) that developed on C3 host plants (Fig. 2). Adults collected at Rock Springs prior to August 4 in 2002 and 2005 (Fig. 1, P1 and P4) had the highest proportion (>25%) of moths that developed on C3 host plants within a population (Fig. 2) suggesting higher proportion of founders to this area were feeding on different host plants prior to immigrating.

### Microsatellite analysis

Significant deviation from HWE occurred for 6 of the 12 loci used (Table 1). Deviation from HWE are nontrivial and need to be appropriately assessed with regard to the overall analysis as they imply genotyping error (Morin et al. 2009), potentially selected loci, or loci linked to selected loci, which do not reflect neutral evolutionary dynamics, including gene flow and drift (Holderegger and Wagner 2006), which are the focus of this study. Additionally, three loci (HzMS4_10, HzMS4_14, and HzMS4_16) had a significant deficit of heterozygotes across all or most populations, suggesting the presence of null alleles at these loci. Two of these loci were also suspected of genotyping error due to stuttering (HzMS4_14 and HzMS_16). These loci were also reported out of HWE and suspected of null alleles and excluded from analyses in previous studies (Perera et al. 2007; Perera and Blanco 2011). Individual removal of each locus, with significant deviation from HWE, from the analysis revealed that the four loci HzMS1_6, HzMS4_10, HzMS4_14, and HzMS4_16 greatly influenced the genetic structure of the *H. zea* populations, and they were not used in subsequent analyses (Table 1). The remaining two loci (HzMS3_41 and HzMS4_23) out of HWE, which were not suspect null alleles, did not influence genetic structure when removed from the analysis and were kept in further analyses (Table 1).

**Table 1:** Summary of locus‐level genetic parameters, number of unique alleles (A), observed heterozygosity (Hobs), expected heterozygosity (Hexp), *F*_ST_ across populations, and whether the loci were included in the final analysis (Included). *F*_ST_ values that significantly differed from HWE expectations based on Monte Carlo permutations are indicated with an asterisk (*)

Locus	GenBank Accession	A	Hobs	Hexp	*F*_ST_	Included
HzMS1_4	EF152205	9	0.326	0.321	0.001	Yes
HzMS3_1	EF152207	6	0.354	0.377	0.003	Yes
HzMS3_11	EF152209	11	0.481	0.514	0.000	Yes
HzMS3_41	EF152210	14	0.390	0.496	0.034*	Yes
HzMS3_48	EF152211	7	0.446	0.518	0.017	Yes
HzMS3_86	EF152212	14	0.068	0.075	0.004	Yes
HzMS4_3	EF152213	8	0.269	0.282	0.006	Yes
HzMS4_23	EF152217	13	0.486	0.622	0.000*	Yes
HzMS1_6	EF152206	21	0.378	0.536	0.122*	No
HzMS4_10	EF152214	14	0.057	0.321	0.013*	No
HzMS4_14	EF152215	14	0.214	0.422	0.001*	No
HzMS4_16	EF152216	9	0.231	0.759	0.016*	No

The eight loci used in the final genetic analyses had no linkage disequilibrium, allelic dropout, or potential null alleles. Mean number of unique alleles per locus was 10.25 (±3.2 SD). Observed heterozygosity ranged from 0.07 to 0.48, while expected heterozygosity ranged from 0.08 to 0.62 (Table 1). Among putative populations, allelic richness ranged from 2.38 to 5.13 (±3.2 SD). The *F*_IS_ ranged from 0.06 to 0.33, with two populations (P1 and P3) not significantly deviating from zero (Table 2), suggesting inbreeding and nonrandom mating across the putative populations. Additionally, the observed *F*_IS_ may have resulted from pheromone traps capturing a mixture of immigrants originated from populations with different allele frequencies. Estimates of *F*_ST_ (Table 2) for all populations ranged between 0.01 and 0.02 and remained unaffected by the removal of individual loci from the analysis. Global *F*_ST_ was 0.01 with an *F*_IS_ of 0.16 and total allelic richness of 82 unique alleles genotyped. Pairwise *F*_ST_ ranged from 0.002 to 0.038 (Table S1).

**Table 2:** Summary of population‐level genetic parameters, number of individuals genotyped (N), mean number of alleles per locus (A), observed heterozygosity (Hobs), expected heterozygosity (Hexp), mean pairwise *F*_ST_, and *F*_IS_ with its 95% confidence interval (*F*_IS_CI)

Population	N	A	Hobs	Hexp	*F*_ST_	*F*_IS_	*F*_IS_CI
P1	30	2.75	0.346	0.386	0.011	0.082	−0.02 to 0.22
P2	64	5.13	0.357	0.421	0.008	0.125	0.01 to 0.28
P3	11	2.38	0.261	0.334	0.016	0.244	−0.13 to 0.52
P4	19	3.38	0.362	0.452	0.014	0.172	0.04 to 0.35
P5	24	3.63	0.302	0.390	0.012	0.207	0.15 to 0.31
P6	52	4.75	0.394	0.422	0.009	0.059	0.00 to 0.14
P7	57	4.25	0.345	0.496	0.008	0.295	0.19 to 0.37
P8	45	4.63	0.342	0.503	0.014	0.329	0.17 to 0.39
P9	48	4.13	0.326	0.422	0.009	0.240	0.12 to 0.31
P10	27	3.13	0.347	0.418	0.013	0.167	0.06 to 0.28
P11	38	3.00	0.327	0.403	0.009	0.156	0.05 to 0.21
P12	95	4.25	0.294	0.427	0.005	0.331	0.16 to 0.39
P13	54	4.63	0.370	0.477	0.008	0.229	0.10 to 0.32

### Genetic structure

**Figure 3:**
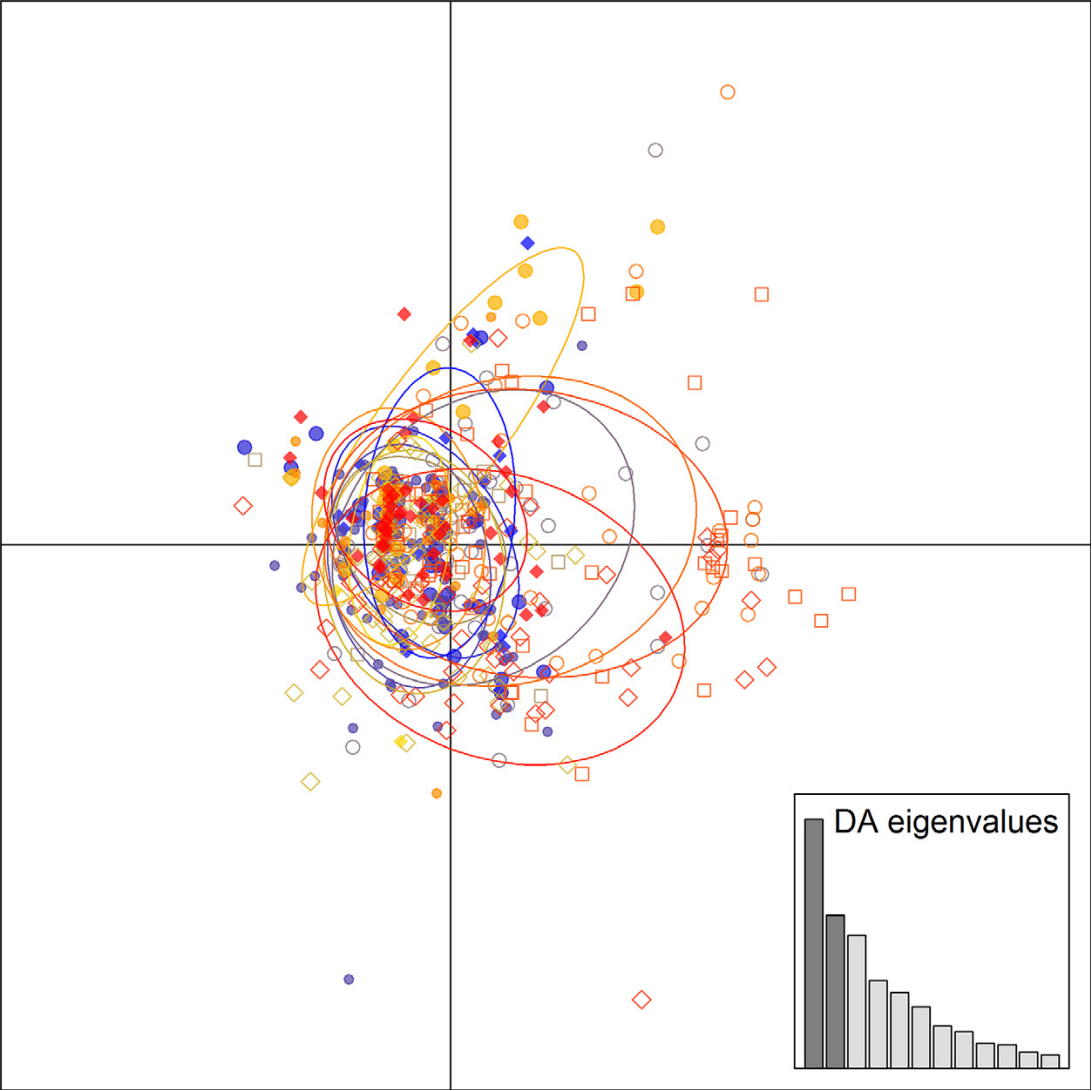
DAPC results using prior assignment of putative population showing the first two axes of the analysis (depicted in the insert plot). Each color and symbol represents a unique population cluster with the corresponding circles showing the prior unique groupings of the 13 putative populations.

Results of the DAPC analysis using prior assignment of putative populations (Fig. 3) revealed very little differentiation among the putative populations. In the nonprior DAPC analysis (Table S2; Fig. S1), *K* = 7 was selected as the most parsimonious clustering based on differences between successive values of BIC summary statistics and successive cluster assignment from repeated DAPC runs (Jombart 2008; Jombart et al. 2010). This additional clustering of *H. zea* individuals predicted using nonprior DAPC, despite showing some variation among individuals, did not correspond to any putative population structure, location, or utilization of C3 or C4 host plant, suggesting that these *H. zea* populations are largely panmictic (Fig S2). Specifically, individuals from each putative population were generally found across all DAPC clusters with a mean of 2 to 14 (SD = 1.89 to 4.22) individuals from each putative population grouping in each of the DAPC cluster. Analysis with STRUCTURE showed even less support for genetic differentiation among populations compared to the DAPC analysis (Fig. S2). STRUCTURE clusters had mean coefficient of ancestry ranging from 0.17 (SD = 0.01) to 0.29 (SD = 0.08) with 28 to 121 individuals assigned to one of seven clusters. Molecular variance analysis (Excoffier and Lischer 2010) of genotype data partitioned by host plant type (C_4_ or C_4_), or by collection site/year, indicated that the greatest molecular variance (>87%) occurred in individuals within the total population. Genetic variation among populations was minimal and not significant (Table S3).

## Discussion

The high genetic diversity and low differentiation observed among the putative populations of *H. zea* sampled suggest that peripheral, northern populations are panmictic with asymmetrical immigration likely dominated by immigrants from southern populations occurring throughout the growing season. That there was no temporal or spatial relationships associated with the putative population clusters suggest immigrants are highly admixed possibly originating from multiple source populations. This tendency is potentially reflected in high *F*_IS_ estimates due to capturing of multiple immigrating populations with differing allelic frequencies per trapping event. The continuous mass pooling and asymmetrical dispersal of *H. zea* is likely influenced by variation in emigration from different southern population generation cycles, which are known to be locally influenced by abiotic (e.g., weather fronts) and biotic (e.g., crop phenology) factors, and occur more frequently in southern (5 to 7 generations/year) than northern (1 to 2 generations/year) regions (Quaintance and Brues 1905; Fitt 1989). Additionally, asymmetrical dispersal may limit selection of *H. zea* to adapt to cooler peripheral habitats, thereby limiting the extent of the over wintering range by counteracting selection of local peripheral populations.

Studies have found generally low genetic variability of *H. zea* across North and South America using allozymes (Sell et al. 1974; Sluss et al. 1978; Mallet et al. 1993; Han and Caprio 2002), mitochondrial DNA (Behere et al. 2007), and microsatellites (Perera and Blanco 2011). However, the population genetic structure of *H. zea* is still largely unknown. Studies of the very closely related *Helicoverpa armigera* (Hübner) suggest that temporal and spatial genetic differentiation may exist in Australia (Scott et al. 2003, 2005), but also showed low genetic differentiation across other larger geographic areas (Nibouche et al. 1998; Zhou et al. 2000; Behere et al. 2007; Endersby et al. 2007) indicating high gene flow among populations. Migration (i.e., long range dispersal) of *H. zea* and other heliothine species is well documented (Hartstack et al. 1982; Farrow and Daly 1987; Gregg et al. 1995), with simulations suggesting that overnight dispersal of a few hundred kilometers is possible (Westbrook 2008; Westbrook and López 2010), and repeated dispersal events enabling semicontinental scale immigration for related noctuid species (Westbrook et al.^2016^).

Temporal structure used in this study is based on previously studied aspects of *H. zea* ecology including lack of adaptation (e.g., cold tolerance) to winter peripheral environments, low number (1 to 2) of generations occurring at peripheral sites, high reproductive rate, and rapid mass dispersal behavior (Quaintance and Brues 1905; Blanchard 1942; Fitt 1989; Morey et al. 2012). Like our findings, a smaller study across southern *H. zea* populations using the same genetic markers (Perera and Blanco 2011) found greater genetic diversity and similarly lower genetic differentiation and expected heterozygosity. Both findings fit well with the highly dispersive ecology of *H. zea* and suggest that peripheral seasonal populations harbor more genetic variation compared to southern populations where continuous selective pressures, such as insecticides, disease, and predation, may reduce genetic variation compared to peripheral populations, which are likely admixtures of immigrants from a much larger geographic area. Previous expectations are that peripheral populations go extinct annually, due to lower temperatures reducing survivability (Fitt 1989). However, recent studies suggest return southward migration of *H. zea* is possible (Pair et al. 1987; Gould et al. 2002), but yet to be confirmed by genetic or mark recapture approaches. *Agrotis ipsilon* (Hufnagel), another long distance migratory lepidopteran species, does utilize southerly airflows to return migrate to the Gulf Coast of the USA from the northern USA each autumn (Showers et al. 1993). Peripheral populations of *H. zea*, resulting from progeny of earlier immigrating populations, may therefore provide sources of genetic diversity and increase admixture with southern populations through return migration from northern populations, thus maintaining migrant (e.g., seasonally invasive) phenotypes (Bock et al. 2015).

*Helicoverpa armigera* is morphologically and phylogenetically closely related to *H. zea*. Recently, *H. armigera* became established in Brazil and is presumed to have originated from Europe (Tay et al. 2013; Murúa et al. 2014). There are severe economic concerns regarding the expansion of *H. armigera* as it is known to develop resistance to insecticides, which are currently being used to manage *H. zea* (Behere et al. 2007; Yang et al. 2013). Similarity in physiology (e.g., diapause) and population genetics between *H. zea* and *H. armigera* indicate that our findings could have direct implications regarding a hypothesis of delayed invasion of *H. armigera* from South America to North America, whereby low genetic diversity of *H. zea* may be limiting successful local adaptation (Kriticos et al. 2015). However, successful establishment is likely to occur, given the favorable climate, agricultural crop flora and landscape present across much of the USA (Kriticos et al. 2015). Overall, understanding the patterns and underlying mechanisms of gene flow in migrant populations is important for developing control strategies for invasive species, such as *H. armigera*, which are expected to be substantially cheaper compared to projected management cost (Kriticos et al. 2015).

In summary, this study revealed that peripheral northern populations of *H. zea* possess high genetic diversity but low genetic differentiation among immigrant populations being invaded from several highly admixed source populations. Additionally, our findings support previous work suggesting that migratory *H. zea* are capable of dispersal over a large geographic range, likely resulting in genetic homogenization and panmixia (Behere et al. 2007; Perera and Blanco 2011). We highlight the general importance of peripheral populations to accumulate genetic diversity and suggest peripheral populations as potential sources for assessing genetic variability for the larger geographic area. In conclusion, it is likely that the previously documented high dispersal ability of *H. zea* and asymmetrical immigration is influencing the high genetic diversity (compared to previous studies) and low genetic differentiation observed. This process could have direct implications for management practices and future assessment of *H. zea* ecology at range limits likely to be affected as climate change is expected to extend species ranges northward due to increased mean annual temperatures.

## Data Accessibility

Microsatellite genotype data and pertinent metadata used in this manuscript have been included in the supplementary Data file S01. Accession numbers of previously published microsatellite loci used in this analysis are listed in Table 1.

## Conflict of Interest

None declared.

## Supporting information


**Figure S1.** Simulation saturation for the allelic richness.
**Figure S2.** DAPC results using no prior assignment of putative population showing the first two axes of the analysis (depicted in the insert plot).
**Figure S3.** Outcome from STRUCTURE analysis assuming *K* = 7. Each vertical bar represents a unique individual (*x*‐axis) with their corresponding assignment score (*y*‐axis) for inclusion in a particular potential population (color).

**Table S1.** Pairwise *F*_ST_ estimates (upper triangle) for putative population pairs.
**Table S2.** Summary of population level genetic parameters for the non‐prior DAPC analysis; the non‐prior populations cluster (Pop), number of individuals genotyped (N), mean number of alleles/locus (A), observed heterozygosity (Hobs), expected heterozygosity (Hexp), *F*_ST_ and *F*_IS_ with its 95% confidence interval (*F*_IS_CI).
**Table S3.** The results of AMOVA of *Helicoverpa zea* populations grouped by collection time to calculate Φ_ST_ (between populations when there was only one group), Φ_CT_ (among groups), or Φ_SC_ (among populations within groups).

**Data S1.** Microsatellite genotypes and collection data for *Helicoverpa zea* adults collected from Landisville and Rock Springs, PA. Size of each microsatellite allele (in Bp) in an individual is given in two consecutive sets of 3‐digits in the 6‐digit genotype.
