# Supplementary material for: Peripheral genetic structure of Helicoverpa zea indicates asymmetrical panmixia
Source: Ecol Evol. 2016 Apr 6;6(10):3198–207. doi: 10.1002/ece3.2106 (PMC4829043; doi:10.1002/ece3.2106)

**Supplementary material (Supplementary Figures S1-S3)**

Peripheral genetic structure of *Helicoverpa zea* indicates assymetrical panmixia

Mathew Seymour, Omaththage P. Perera, Howard W. Fescemyer, Ryan E. Jackson, Shelby J. Fleischer, Craig A. Abel

Figure S1. Simulation saturation for the allelic richness. Each panel shows a unique putative population site (see Fig 1). Each point is
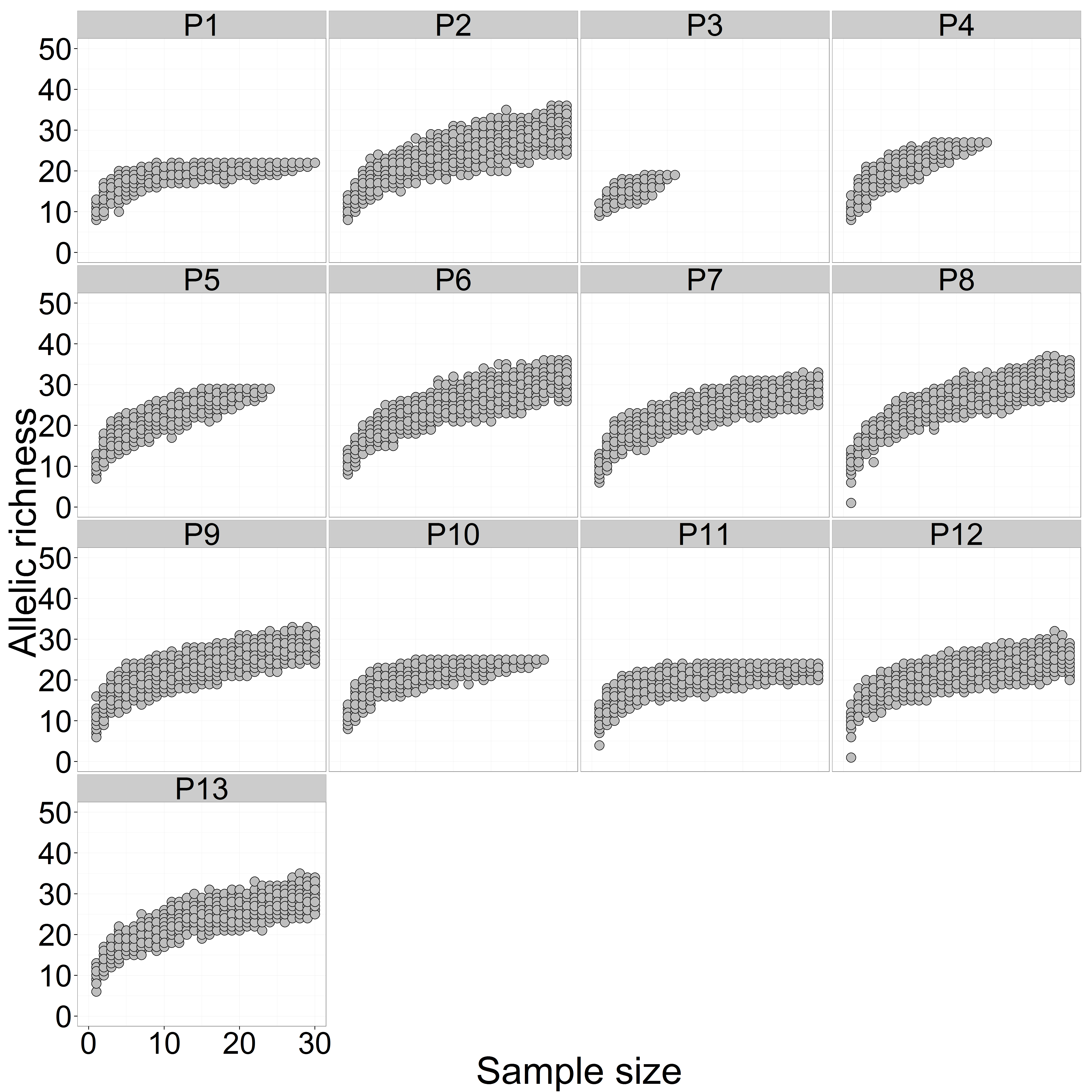
a single simulation run with 100 iterations per sample size.

Figure S2. DAPC results using no prior assignment of putative population showing the first two axes of the analysis (depicted in the insert plot). Each color represents a unique population cluster (K = 7; based on change in BIC scores and parsimony).


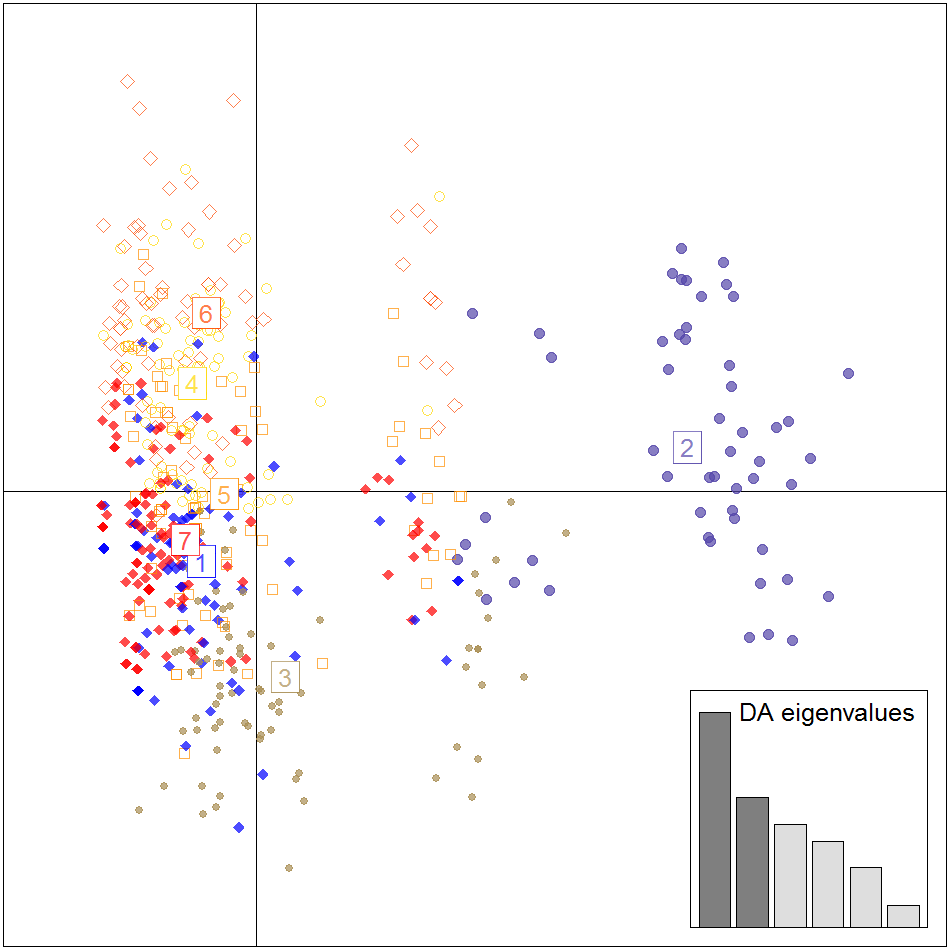


Figure S3. Outcome from STRUCTURE analysis assuming K=7. Each vertical bar represents a unique individual (x-axis) with their corresponding assignment score (y-axis) for inclusion in a particular potential population (color). .


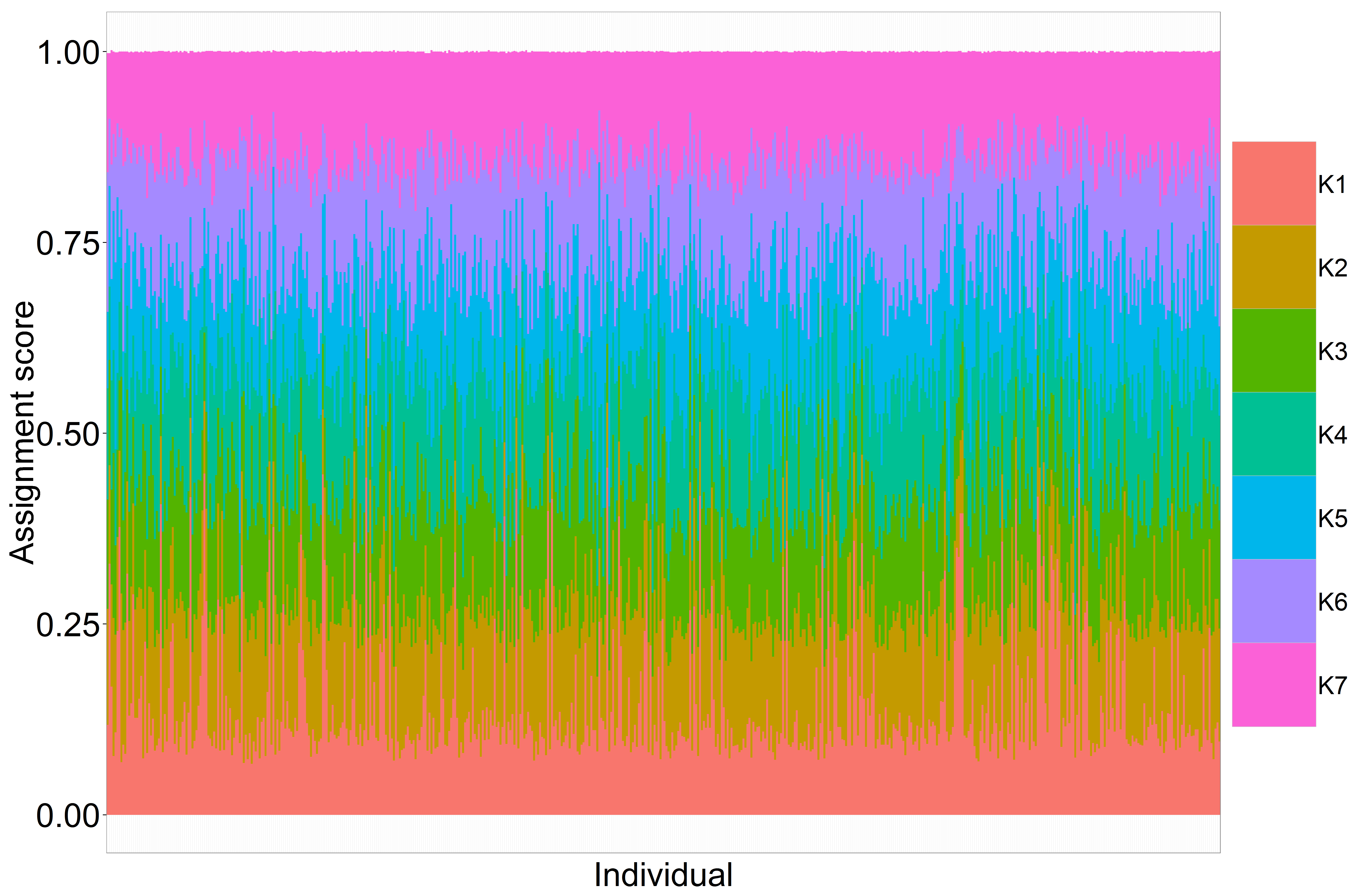

Supplement: Supplementary file 1 — Figure S1. Simulation saturation for the allelic richness. Figure S2. DAPC results using no prior assignment of putative population showing the first two axes of the analysis (depicted in the insert plot). Figure S3. Outcome from STRUCTURE analysis assuming K = 7. Each vertical bar represents a unique individual (x‐axis) with their corresponding assignment score (y‐axis) for inclusion in a particular potential population (color). [file ECE3-6-3198-s001.doc]
