# Supplementary material for: Peripheral genetic structure of Helicoverpa zea indicates asymmetrical panmixia
Source: Ecol Evol. 2016 Apr 6;6(10):3198–207. doi: 10.1002/ece3.2106 (PMC4829043; doi:10.1002/ece3.2106)
Supplement: Supplementary file 2 — Table S1. Pairwise F ST estimates (upper triangle) for putative population pairs. Table S2. Summary of population level genetic parameters for the non‐prior DAPC analysis; the non‐prior populations cluster (Pop), number of individuals genotyped (N), mean number of alleles/locus (A), observed heterozygosity (Hobs), expected heterozygosity (Hexp), F ST and F IS with its 95% confidence interval (F ISCI). Table S3. The results of AMOVA of Helicoverpa zea populations grouped by collection time to calculate ΦST (between populations when there was only one group), ΦCT (among groups), or ΦSC (among populations within groups). [file ECE3-6-3198-s002.doc]

**Supplementary material (Tables S1 –S3)**

Peripheral genetic structure of *Helicoverpa zea* indicates assymetrical panmixia

Mathew Seymour, Omaththage P. Perera, Howard W. Fescemyer, Ryan E. Jackson, Shelby J. Fleischer, Craig A. Abel

Table S1. Pairwise F_ST_ estimates (upper triangle) for putative population pairs.

|  |  |  |  |  |  |  |  |  |  |  |  |  |  |
| --- | --- | --- | --- | --- | --- | --- | --- | --- | --- | --- | --- | --- | --- |
|  | P1 | P2 | P3 | P4 | P5 | P6 | P7 | P8 | P9 | P10 | P11 | P12 | P13 |
| P1 | 0.000 | 0.014 | 0.028 | 0.020 | 0.010 | 0.011 | 0.008 | 0.016 | 0.011 | 0.006 | 0.008 | 0.007 | 0.008 |
| P2 | - | 0.000 | 0.009 | 0.011 | 0.013 | 0.008 | 0.005 | 0.013 | 0.005 | 0.014 | 0.009 | 0.002 | 0.006 |
| P3 | - | - | 0.000 | 0.014 | 0.024 | 0.010 | 0.014 | 0.025 | 0.009 | 0.038 | 0.020 | 0.006 | 0.013 |
| P4 | - | - | - | 0.000 | 0.017 | 0.014 | 0.014 | 0.025 | 0.010 | 0.023 | 0.017 | 0.007 | 0.009 |
| P5 | - | - | - | - | 0.000 | 0.010 | 0.013 | 0.019 | 0.011 | 0.013 | 0.006 | 0.006 | 0.011 |
| P6 | - | - | - | - | - | 0.000 | 0.004 | 0.012 | 0.007 | 0.014 | 0.008 | 0.006 | 0.009 |
| P7 | - | - | - | - | - | - | 0.000 | 0.010 | 0.006 | 0.008 | 0.006 | 0.004 | 0.006 |
| P8 | - | - | - | - | - | - | - | 0.000 | 0.022 | 0.012 | 0.013 | 0.012 | 0.013 |
| P9 | - | - | - | - | - | - | - | - | 0.000 | 0.014 | 0.007 | 0.003 | 0.006 |
| P10 | - | - | - | - | - | - | - | - | - | 0.000 | 0.009 | 0.008 | 0.008 |
| P11 | - | - | - | - | - | - | - | - | - | - | 0.000 | 0.004 | 0.008 |
| P12 | - | - | - | - | - | - | - | - | - | - | - | 0.000 | 0.004 |
| P13 | - | - | - | - | - | - | - | - | - | - | - | - | 0.000 |

Table S2. Summary of population level genetic parameters for the non-prior DAPC analysis; the non-prior populations cluster (Pop), number of individuals genotyped (N), mean number of alleles/locus (A), observed heterozygosity (Hobs), expected heterozygosity (Hexp), F_ST_ and F_IS_ with its 95% confidence interval (F_IS_CI).

| Pop | N | A | Hobs | Hexp | F_ST_ | F_IS_ | F_IS_CI |
| --- | --- | --- | --- | --- | --- | --- | --- |
| NP1 | 92 | 6.38 | 0.25 | 0.29 | 0.11 | 0.14 | -0.02-0.29 |
| NP2 | 75 | 5.88 | 0.36 | 0.39 | 0.09 | 0.04 | -0.01-0.13 |
| NP3 | 57 | 6.38 | 0.38 | 0.43 | 0.08 | 0.16 | 0.02-0.28 |
| NP4 | 72 | 6.13 | 0.4 | 0.4 | 0.09 | 0.03 | -0.2-0.18 |
| NP5 | 103 | 5.50 | 0.31 | 0.34 | 0.09 | 0.17 | -0.05-0.26 |
| NP6 | 103 | 6.13 | 0.33 | 0.35 | 0.08 | 0.06 | -0.05-0.15 |
| NP7 | 62 | 5.63 | 0.42 | 0.41 | 0.1 | 0.03 | -0.26-0.2 |

Table S3. The results of AMOVA of *Helicoverpa zea* populations grouped by collection time to calculate Φ_ST_ (between populations when there was only one group), Φ_CT_ (among groups), or Φ_SC_ (among populations within groups). Populations grouped together in each analysis are shown within square brackets. P1-P13 are populations used in DAPC analysis; R02 and R05 are 2002 and 2005 collections, respectively, from Rocksprings, PA; L2005 is the 2005 collection from Landisville, PA.

Grouping Variance component % Total variance Φ-statistic p

1. All populations

Group 1 [All populations] Among populations 0.55 Φ_ST_ = 0.00548 =0.02248

2. Grouped relative to capture peak

Group 1 [P1, P4, P5, P10, P11] Among groups 0.22 Φ_CT_ =-0.00219 =0.06354

Group 2 [P6, P7, P12] Among populations 0.40 Φ_SC_ =-0.00399 =0.10850

Group 3 [P2, P3, P8, P9,P13]

3. Grouped by Collection site and year

Group 1 [R02 and R05] Among groups 0.55 Φ_CT_ = 0.00012 =0.68035

Group 2 [L05] Among populations -0.11 Φ_SC_ = 0.00110 =0.68328

4. Grouped by Host plant type

Group 1 [C3, C4] Among populations -0.27 Φ_ST_ = -0.00272 =0.99902
